# Functional Development of Principal Neurons in the Anteroventral Cochlear Nucleus Extends Beyond Hearing Onset

**DOI:** 10.3389/fncel.2019.00119

**Published:** 2019-03-28

**Authors:** Maria Katharina Müller, Sasa Jovanovic, Christian Keine, Tamara Radulovic, Rudolf Rübsamen, Ivan Milenkovic

**Affiliations:** ^1^Carl Ludwig Institute for Physiology, Faculty of Medicine, University of Leipzig, Leipzig, Germany; ^2^Carver College of Medicine, Department of Anatomy and Cell Biology, University of Iowa, Iowa City, IA, United States; ^3^Institute of Biology, Faculty of Life Sciences, University of Leipzig, Leipzig, Germany; ^4^School of Medicine and Health Sciences, Carl von Ossietzky University Oldenburg, Oldenburg, Germany

**Keywords:** cochlear nucleus, bushy cells, stellate cells, *in vivo*, mouse

## Abstract

Sound information is transduced into graded receptor potential by cochlear hair cells and encoded as discrete action potentials of auditory nerve fibers. In the cochlear nucleus, auditory nerve fibers convey this information through morphologically distinct synaptic terminals onto bushy cells (BCs) and stellate cells (SCs) for processing of different sound features. With expanding use of transgenic mouse models, it is increasingly important to understand the *in vivo* functional development of these neurons in mice. We characterized the maturation of spontaneous and acoustically evoked activity in BCs and SCs by acquiring single-unit juxtacellular recordings between hearing onset (P12) and young adulthood (P30) of anesthetized CBA/J mice. In both cell types, hearing sensitivity and characteristic frequency (CF) range are mostly adult-like by P14, consistent with rapid maturation of the auditory periphery. In BCs, however, some physiological features like maximal firing rate, dynamic range, temporal response properties, recovery from post-stimulus depression, first spike latency (FSL) and encoding of sinusoid amplitude modulation undergo further maturation up to P18. In SCs, the development of excitatory responses is even more prolonged, indicated by a gradual increase in spontaneous and maximum firing rates up to P30. In the same cell type, broadly tuned acoustically evoked inhibition is immediately effective at hearing onset, covering the low- and high-frequency flanks of the excitatory response area. Together, these data suggest that maturation of auditory processing in the parallel ascending BC and SC streams engages distinct mechanisms at the first central synapses that may differently depend on the early auditory experience.

## Introduction

The auditory system decodes complex natural sounds by analyzing the frequency, amplitude and temporal information to master the tasks like sound source localization and discrimination of acoustic objects with extraordinary precision. Following the sensory signal transduction by the inner hair cells (IHCs), sound information is transformed into discrete action potentials, conveyed through the auditory nerve fibers (ANF) (Fekete et al., 1984; Brown and Ledwith, 1990; Liberman, 1991). The ANF inputs segregate into ascending neuronal pathways specialized in parallel processing of different acoustic features (Joris and Yin, 1992; Zatorre et al., 1992; Romanski et al., 1999; Belin and Zatorre, 2000; Smith and Spirou, 2002; van der Heijden and Joris, 2003; Oertel and Young, 2004; Yao et al., 2015).

Primary auditory nerve inputs make central synapses in the anteroventral cochlear nucleus onto (among others) the two principal neurons, bushy cells (BCs) and T-stellate cells (further referred to as SCs). They contribute to processing in different auditory pathways: (i) BCs preserve the temporal structure of sound which is crucial for sound source localization in the superior olivary complex (Young et al., 1988; Joris et al., 1994); and (ii) SCs encode the dynamic amplitude profile of sound signals (Blackburn and Sachs, 1990; Frisina et al., 1990) and provide input to the contralateral inferior colliculus (Cant and Benson, 2003). Despite their common input through auditory nerve, the specific roles of BCs and SCs in auditory processing are accomplished through distinct quantity and morphology of respective inputs (Brawer and Morest, 1975; Cao and Oertel, 2010), different morphological and biophysical properties (Oertel, 1983; Manis and Marx, 1991), and diverging ascending projections (Cant and Benson, 2003). To date, the *in vivo* functional development of BCs and SCs in mice is still not well understood. Our knowledge about the cochlear nucleus development is based on data from acute slice preparations from both low-frequency hearing animals (chick: Lawrence and Trussell, 2000; Brenowitz and Trussell, 2001; Lu and Trussell, 2007; Tang et al., 2013; Goyer et al., 2015; Sanchez et al., 2015; Hong et al., 2016; Oline et al., 2016; gerbil: Milenković et al., 2007; Witte et al., 2014; Jovanovic et al., 2017; Nerlich et al., 2017) and high-frequency hearing animals (rat: Bellingham et al., 1998; mouse: Wu and Oertel, 1987; Lu et al., 2007; Yang and Xu-Friedman, 2010; Campagnola and Manis, 2014). Respective *in vivo* developmental data were collected more than 30 years ago from the cochlear nucleus of chicken (Saunders et al., 1973; Rubel and Parks, 1975), gerbil (Woolf and Ryan, 1985), and cat (Pujol, 1972; Romand and Marty, 1975; Brugge et al., 1978). Expanding the use of transgenic mice in auditory research increases the importance of revealing the developmental time course of auditory processing in the cochlear nucleus.

Here, we characterized the maturation of spontaneous and acoustically evoked activity in BCs and SCs between the hearing onset (P12; Sonntag et al., 2009) and young adulthood (P30) of CBA/J mice. The present results reveal functionally immature neuronal response properties at hearing onset with cell-type specific maturation patterns during the early auditory experience.

## Materials and Methods

All experimental procedures were approved by the Saxonian District Government Leipzig (TVV 20/14, T34/16) and conducted according to the European Communities Council Directive (86/609/EEC). *In vivo* recordings were performed from the AVCN of 20 CBA/J mice (Janvier Labs, Le Genest-Saint-Isle, France) of either sex, bred in the animal facility of the Institute of Biology, Faculty of Life Sciences of the University of Leipzig. The development of spontaneous and acoustically evoked activity in AVCN units was assessed at five time points between hearing onset and young adulthood (3–5 animals per age group at postnatal days (P) 12, 13, 14, 18, and 30). Slice recordings were conducted in P10–18 mice of either sex.

### Surgical Preparation

For surgical preparation, animals were anesthetized with an initial intraperitoneal injection of a mixture of ketamine hydrochloride (0.1 mg/g body weight; Ketamin-Ratiopharm, Ratiopharm) and xylazine hydrochloride (5 μg/g body weight; Rompun, Bayer). Throughout recording sessions, anesthesia was maintained by additional subcutaneous application of one-third of the initial dose every 60–120 min, depending on the animal’s age. Animals were fixed in a stereotaxic frame using a brass bolt and the AVCN was targeted dorsally through a hole in the skull as described previously (Kopp-Scheinpflug et al., 2002).

### Acoustic Stimulation

Recordings were performed in a sound-attenuating chamber (Type 400, Industrial Acoustic Company, North Aurora, IL, USA) with the animal stabilized in a custom-made stereotaxic apparatus positioned on a vibration-isolated table. Animal’s temperature was kept at 37°C with a feedback-controlled heating pad. Acoustic stimuli were digitally generated using custom-written Matlab functions (version 7.5, The MathWorks Inc, Natick, MA, USA, RRID:SCR_001622). The stimuli were transferred to a D/A converter (RP2.1 real-time processor, 97.7 kHz sampling rate, Tucker-Davis Technologies, Alachua, FL, USA) and delivered through custom-made earphones (acoustic transducer: DT 770 pro, Beyer Dynamics) fitted with plastic tubes (length 35 mm, diameter 5 mm) which were positioned in the outer ear canal ~4 mm in front of the eardrum.

### Stimulus Protocol and Data Acquisition

Juxtacellular recordings of AVCN single-units were performed with glass micropipettes (GB150F-10, Science Products, 5–10 MΩ) filled with 3 M KCl. Four protocols were used for acoustic stimulation: (i) pure tone pulses (100 ms duration, 5 ms cos^2^ rise-fall time, 200 ms inter-stimulus interval) derived from a predefined matrix of frequency/intensity pairs (20 frequencies on a logarithmic scale, 10 intensity levels on a linear scale, 4–5 repetitions per frequency-intensity combination) were presented in a pseudo-random manner to compute the frequency response areas (Englitz et al., 2009); (ii) temporal response properties were measured during repetitive presentation of pure tones (100 ms duration, 5 ms cos^2^ rise-fall time, 300 or 1,000 ms inter-stimulus interval, 150 repetitions) at the units’ individual characteristic frequency (CF) 20 dB above response threshold (Sonntag et al., 2009; Keine et al., 2016); (iii) temporal encoding of fluctuations in sound amplitude was quantified from the responses to sinusoidal amplitude-modulated (SAM) tone bursts (200 ms duration, 300 ms inter-stimulus interval, 150 repetitions) presented at a CF, 20 dB above threshold. The modulation depth was 100% and five modulation frequencies were used: 20, 50, 100, 200, and 500 Hz; (iv) spontaneous activity was acquired in the absence of acoustic stimulation (recording time 1–5 min, depending on the unit’s firing rate) and used to calculate the average spontaneous firing rate, coefficient of variation (CV_spont_) of interspike intervals (ISIs) and to generate a mean waveform of the recorded voltage signals (Sonntag et al., 2009).

The recording sites were histologically verified by Fluorogold. The position of the last recorded unit at the end of the experiment was approached according to its stereotaxic coordinates with a new electrode containing fluorogold and the dye was iontophoretically injected for 5 min with a current of 5 μA. Subsequently, the animal was perfused transcardially with 0.9% NaCl solution followed by 5% PFA. Coronal slices containing the cochlear nucleus were cut on a vibratome (HM 650V, Microm) and the tissue sections (50 μm thick) were visualized under a fluorescent microscope (Zeiss Axioskop 2).

### Data Analysis

Recorded voltage signals were amplified (Neuroprobe 1600, A-M Systems), digitized at a sampling rate of 97.7 kHz (RP2.1, Tucker-Davis Technologies, Alachua, FL, USA), bandpass filtered (50–7,000 Hz) using a zero-phase forward and reverse digital IIR filter, and stored for offline analysis using custom-written Matlab functions (Dietz et al., 2012; Typlt et al., 2012; Keine et al., 2016; Jovanovic et al., 2017). Recordings were required to meet three criteria: (i) signal-to-noise ratio of at least 8:1 (dataset: 15.7 ± 8.2, *n* = 136); (ii) fluctuations of the spike height measured as standard deviation (SD)/mean not exceeding 20% (dataset: 11.3 ± 3.8%, *n* = 136); and (iii) the recorded signals showed a stable, uniform waveform. In P18–30 mice, BCs were identified by their complex waveform allowing differentiation of the prepotential (AP of the endbulb of Held terminal, PP), the excitatory postsynaptic potential (EPSP) and the postsynaptic AP, and by the primary-like peristimulus time histogram (PSTH; Pfeiffer, 1966; Young et al., 1988; Blackburn and Sachs, 1989; Englitz et al., 2009; Typlt et al., 2010, 2012). SCs of the same age were identified by their biphasic AP waveform and “chopper” PSTH (Rhode and Smith, 1986; Young et al., 1988; Typlt et al., 2012). The immature cells recorded from P12–14 mice, however, rarely showed chopper-type PSTHs or waveforms with prepotentials, rendering the classical separation inadequate. Thus, immature units were distinguished by hierarchical clustering based on Euclidean square distance to evaluate the separation of AP repolarization slopes between the two cell types (see “Results” section). Cluster results were evaluated based on silhouette values, a measure of object cohesion within a cluster and an indicator of separation between clusters (Rousseeuw, 1987). Silhouette values range from −1 to 1 with high values indicating a good separation between clusters.

From in total 27 P18 and P30 units classified as SCs, only two had onset chopper responses to acoustic stimulation, consistent with D-stellate or radiate multipolar cells (Smith and Rhode, 1989; Winter and Palmer, 1995; Palmer et al., 2003). These units were excluded from the analysis because we aimed at transient or sustained chopper units, i.e., T-SCs. In SCs with inconclusive PSTH, D-SCs can be distinguished according to their broad tuning (Smith and Rhode, 1989). The SCs included in the study had neither onset chopper responses nor broad tuning typical for D-SCs.

#### Frequency Response Area

The excitatory response area of a unit was defined as a field of frequency/intensity combinations that elicited a significant increase in AP discharge rate above the spontaneous rate (*p* < 0.01). Excitatory response areas were used to determine the response threshold (the lowest stimulus intensity resulting in an increase of AP spiking) and CF (the sound frequency causing increased firing at the lowest sound intensity). Threshold/CF pairs for individual cells were used to construct quadratic polynomial trend curves for each age group and cell type (Castellote et al., 2014) to derive an estimate of the cell type based audiogram. The maximum discharge rate was determined as the maximum number of APs per second elicited by one of the 200 frequency/intensity pairs. The relative bandwidth of the excitatory field (Q_n_) was calculated as the ratio between the unit’s CF and the frequency bandwidth (CF/BW) at *n* = 10, 20, and 30 dB above the response threshold. Rate-level function (RLF) at a CF and half an octave below CF (CF/1.4), were determined by averaging the discharge rates obtained with 4–5 repetitions at each sound pressure level. The dB range between 10 and 90% of the rising slope of the rate level function at CF was defined as the dynamic range. For units with prominent inhibitory sidebands, indicated by a significant decrease in AP firing below the spontaneous rate, the inhibitory sideband threshold, frequency (F_inh_), and relative bandwidth were calculated, using the same approach as for the analogous parameters of the excitatory receptive field. In addition, the strength of inhibition was estimated as the relative reduction of spontaneous firing rate at 20 dB above the inhibitory threshold. In units with sufficient spontaneous rates (>8 AP/s), the rate level functions were also calculated at F_inh_.

#### PSTH and SAM

PSTHs were used to determine the first spike latency (FSL), calculated as the time between stimulus onset and the peak of a kernel density function (Botev et al., 2010) fitted over the AP spike times. The coefficient of variation of ISIs (CV_PSTH_ = SD_ISI_/mean ISI) was calculated for a 10 ms window starting at FSL-SD_FSL_.

The classification of PSTHs in P18 and P30 mice was done according to criteria described earlier, which are based on the shape and CV_PSTH_ values (Rhode and Smith, 1986; Young et al., 1988; Blackburn and Sachs, 1989; Roos and May, 2012). Two types of temporal response patterns were acquired during our recordings: (i) PSTH, with phasic-tonic time course and CV_PSTH_ > 0.4, characteristic for BCs; (ii) chopper PSTH displaying at least three regularly spaced periods of increased spiking probability and CV_PSTH_ < 0.4, characteristic for SCs in the AVCN. Additional PSTH recordings with 1 s inter-stimulus interval were acquired from BCs to measure the recovery of spontaneous activity following the post-stimulus depression after the offset of sound stimulation. In each unit, the recovery phase of spontaneous activity was normalized and fitted with mono- and bi-exponential function. The weighted time constant was calculated as τ_wd_ = f × τ_fast_ + (1 − f) × τ_slow_ where τ_fast_ and τ_slow_ are the fast and slow time constants, respectively and *f* is the relative contribution of τ_fast_. An *F*-test was used to statistically determine the better fit (Mandel, 1964). The F-ratio was calculated according to the equation:

$$F=\frac{\left(SS_{\text{mono}}-SS_{\text{bi}})\times (n-p_{\text{bi}}-1\right)}{\left(SS_{\text{bi}}\times (p_{\text{bi}}-p_{\text{mono}})\right)},$$

where *SS*, *n*, and *p* are residual sum of squares, number of data points, and number of model parameters, respectively. The *F*-value was used to determine the *p*-value from an F-distribution with *p*_2_-*p*_1_ and *n-p*_2_ degrees of freedom. The bi-exponential model was chosen over the mono-exponential model at *p* < 0.05.

Recordings during SAM stimulation were used to generate peristimulus time- and period-histograms. The first 20 ms of each stimulus-triggered recording were discarded to reduce onset effects. The precision of AM coding by neuronal activity was assessed by calculating the vector strength (VS; Goldberg and Brown, 1969) of spike discharges. Modulation depth of the neuronal response and between-trial reproducibility of neuronal activity during SAM stimulation were calculated as described previously (Joris et al., 2006; Keine et al., 2016). In brief, the reproducibility was estimated from the central peak of the within-cell between-trial cross-correlation (or shuffled autocorrelation) for identical stimulus presentations. If the neuronal discharge follows the periodic stimulus, the correlogram of spike discharges is itself periodic and the degree of firing rate modulation can be estimated from the SD of the first cycle of this correlogram.

#### Spontaneous Activity and Waveform Analysis

The neurons’ spontaneous activity during the absence of acoustic stimulation was used to capture the average extracellular waveform. In BCs, the following physiologically relevant parameters were quantified: (i) the synaptic transmission delay defined as the time between the positive peaks of the presynaptic signal component and the postsynaptic AP (prepotential-AP delay); and (ii) the duration of the postsynaptic AP, quantified as the time between the peak of the postsynaptic AP and the first local minimum during the subsequent repolarization phase.

During initial auditory experience (≥P12), spontaneous activity in some units resembles a combination of bursting pattern, typically seen in the first postnatal week, and Poisson-like spiking, found in animals few days after hearing onset (Jones et al., 2007; Sonntag et al., 2009). The histograms showing the probability density of interspiking intervals in Figure 2A were fitted with a Gaussian mixture distribution by maximum likelihood, using the Expectation-Maximization (EM) algorithm (Matlab function fitgmdist). The optimal number of components was determined using the Bayes information criterion.

**Figure 1:**
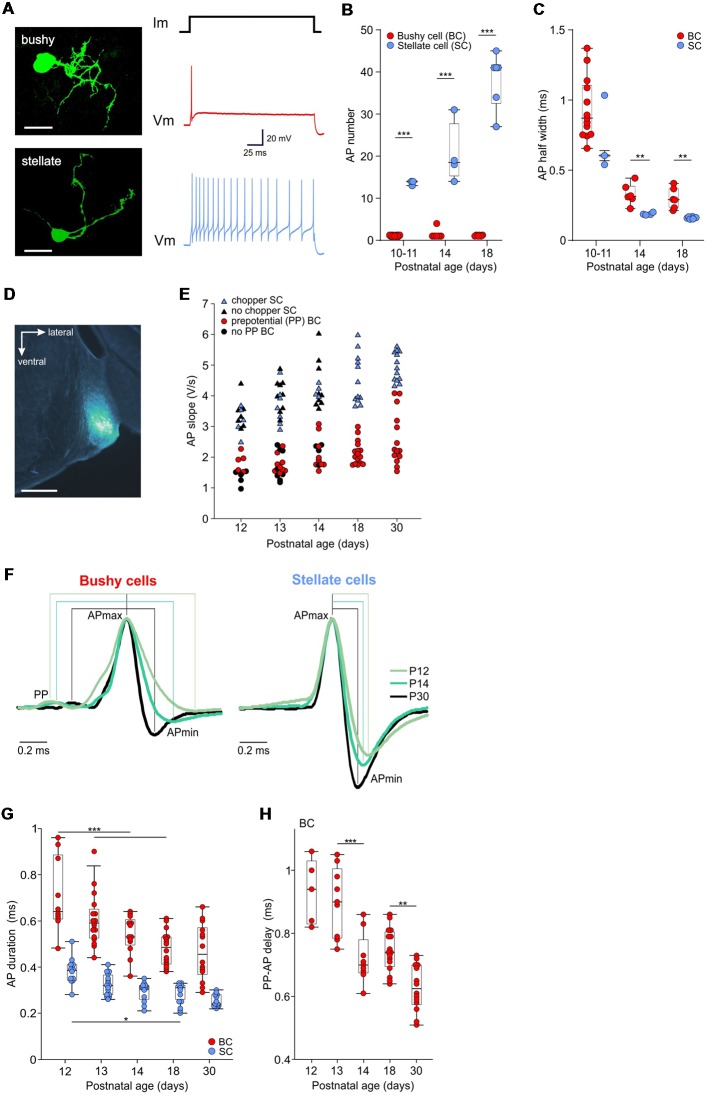
Development of action potential properties in bushy cells (BCs) and stellate cells (SCs). (**A**; Left) Biocytin labeling of recorded neurons reveals the BC (top) and SC (bottom) morphology, both from P14 (scale bar 25 μm). The recordings to the right, acquired from the respective cells, show the phasic firing in response to a depolarizing current step in the BC, and tonic firing in the SC. **(B)** The number of APs elicited by a suprathreshold current injection. Dots show the mean values for each cell obtained from >10 repetitions. Note that BCs (red) mostly generate a single AP, irrespective of age. The number of APs in SCs (blue) increases with age [the effect of age *p* < 0.001, two-way ANOVA]. Box-plots show medians with 25 and 75 percentiles, and interdeciles. **(C)** The AP half-width is shown as the mean value for each BC (red dots) and SC (blue dots; P10–11 BC *n* = 13, SC *n* = 3; P14 BC *n* = 6, SC *n* = 4; P18 BC *n* = 6, SC *n* = 6; effect of cell type *p* = 0.015, effect of age *p* < 0.001, interaction cell type × age *p* = 0.94; two-way ANOVA). **(D)** The *in vivo* recording position, verified by an iontophoretic injection of fluorogold. The image shows a part of a coronal slice containing the anteroventral cochlear nucleus labeled in a P14 mouse (scale bar 500 μm). **(E)** The repolarization slope values calculated from juxtacellular AP recordings *in vivo*. Red dots depict units with prepotentials (PP), i.e., BCs. Blue triangles show units with chopper firing pattern corresponding to SCs. The remaining immature units showing neither PP nor chopper firing pattern (black symbols) separate according to their slope values in the two populations. **(F)** Mean waveforms of extracellular APs recorded in BC (left) and SC (right) at P12, P14, and P30, normalized to and aligned at the AP maximum. Each waveform represents the average AP time course during spontaneous activity (P12: BC *n* = 1,274, SC *n* = 649; P14: BC *n* = 1,055, SC *n* = 1,251; P30: BC *n* = 2,524, SC *n* = 3,062). Discernible PP prior to the AP are characteristics of BCs, while absent in SCs. **(G)** Population data showing AP shortening with age and generally longer APs in BCs than in SCs (effect of cell type *p* < 0.001, effect of age *p* < 0.001, interaction cell type × age *p* = 0.97; two-way ANOVA). **(H)** Transmission delay at the auditory nerve fiber-BC synapse, measured as time between PP and AP peak (*p* < 0.001, one-way ANOVA; *p < 0.05, **p < 0.01; ***p < 0.001).

**Figure 2:**
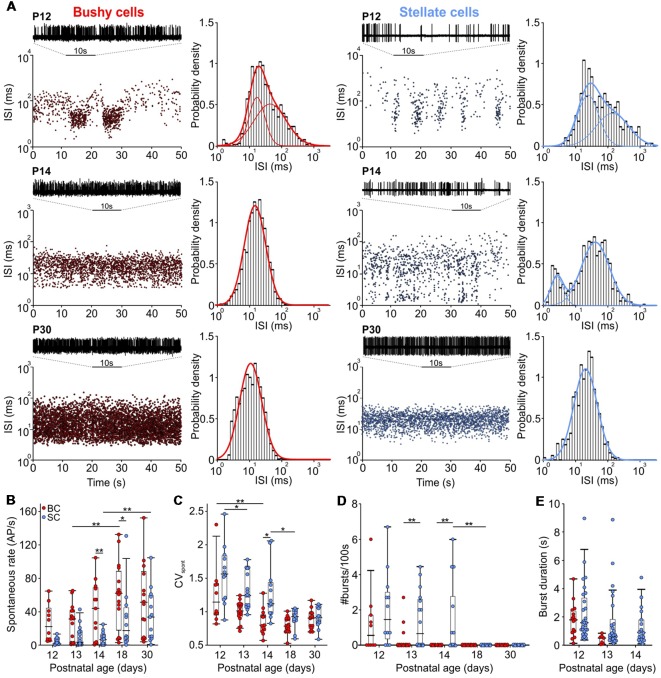
The rate of spontaneous AP activity increased with age, changing from bursting to Poisson-like firing. **(A)** Interspike interval (ISI) distribution over time for BCs (left) and SCs (right) from P12 (upper panels), P14 (middle panels), and P30 (lower panels). In each panel, representative 10 s voltage traces show the succession of AP discharges. Panels to the right show Gaussian fits to the probability of ISIs for the respective BCs and SCs. Note the loss of longer ISIs with maturity and only a single distribution in a P14 BC, as opposed to two distributions in a P14 SC. **(B)** Spontaneous firing rates increased during the development of both cell types (effect of cell type *p* < 0.001, effect of age *p* < 0.001, interaction cell type × age *p* = 0.6; two-way ANOVA). **(C)** Coefficient of variation (CV_spont_) values decreased with development in both cell types being smaller in BCs than in SCs (effect of cell type *p* < 0.001, effect of age *p* < 0.001, interaction cell type × age *p* = 0.05; two-way ANOVA). **(D)** The number of bursts generated during 100 s periods at different ages. Note that only a small portion of BCs still generated bursts at P12 and P13 (effect of cell type *p* = 0.002, effect of age *p* < 0.001, interaction cell type × age *p* = 0.1; two-way ANOVA). **(E)** The duration of bursts decreased similarly between P12 and P13 in both cells types (effect of cell type *p* = 0.07, effect of age *p* = 0.04, interaction cell type × age *p* = 0.44; two-way ANOVA). Note the absence of bursts in P14 BCs (*p < 0.05, **p < 0.01).

To analyze the regularity of spontaneous activity, the CV_spont_ = SD_ISI_/mean ISI, was calculated for all developmental stages. To identify bursts in P12–P14 animals the observed ISIs were compared to a theoretical gamma probability distribution (Hoel et al., 1971; Klenke, 2006). In brief, assuming that AP firing of AVCN neurons can be approximated as a Poisson-like process, then the probability to encounter one ISI in time period τ can be approximated by *P* = 1-e^−λτ^, where λ is the average neuronal firing rate. The probability to encounter *k* ISIs during the time period τ was calculated as *k*-fold convolution of exponential distribution density, thereby yielding a gamma distribution of waiting times for *k* ISIs. The resulting probability is $P=\int_{0}^{\tau }\frac{x^{\text{k}-1}(\lambda^{\text{k}}e^{-\lambda x})}{(k-1)!}\text{d}x$ with *x* indicating an ISI. Based on the statistical analysis of recorded AP times, spike trains where *p* < 0.01 for at least 10 ISIs (*k* ≥ 10) were defined as bursts. This allowed to determine the number of bursts for each cell.

### Slice Preparation

Acute slices of the cochlear nucleus complex were cut in a coronal plane (200 μm) from P10–18 mice of either sex, killed by decapitation (using methods approved by the Saxonian district Government Leipzig). Slicing was done with a vibratome (Microm HM 650) in cold (3–4°C) low-calcium artificial cerebrospinal fluid (ACSF) solution containing (in mM): 125 NaCl, 2.5 KCl, 0.1 CaCl_2_, 3 MgCl_2_, 1.25 NaH_2_PO_4_, 25 NaHCO_3_, 25 glucose, 2 sodium pyruvate, 3 myo-inositol, 0.5 ascorbic acid, continuously bubbled with 5% CO_2_ and 95% O_2_, pH 7.4. Slicing solution contained less Ca^2+^ and more Mg^2+^ than the standard ACSF in order to avoid Ca^2+^-dependent signaling and activation of N-Methyl-D-Aspartate Receptor (NMDAR). After cutting, slices were incubated in the standard recording ACSF containing 125 NaCl, 2.5 KCl, 2 CaCl_2_, 1 MgCl_2_, 1.25 NaH_2_PO_4_, 25 NaHCO_3_, 25 glucose, 2 sodium pyruvate, 3 myo-inositol, 0.5 ascorbic acid, continuously bubbled with 5% CO_2_ and 95% O_2_, pH 7.4, for 30 min at 37°C to clear up the tissue surface and restore cellular processes. Thereafter, slices were stored at room temperature to prolong their usability until recording. Recordings were made at 34 ± 1°C. The data were grouped as: P10–11 (just before hearing onset), P14 (just after hearing onset), and P18 (early auditory experience).

### Whole-Cell Recordings in Acute Slices

Current clamp recordings from BCs and SCs were done as described previously (Dietz et al., 2012). In brief, patch pipettes were made of borosilicate glass (Science Products) with Narishige PC-10 vertical puller to have resistances of 3–5 MΩ when filled with internal solution containing (mM): 130 potassium-gluconate, 10 KCl, 1 NaCl, 0.05 CaCl_2_, 10 HEPES, 0.1 EGTA, 5 mM phosphocreatine, 2 mM ATP disodium salt, 0.3 mM GTP disodium salt (pH 7.3 with KOH). Biocytin (0.2%) was supplemented for labeling of recorded neurons. Current clamp recordings were acquired with Multiclamp 700B amplifier (Molecular Devices) using bridge balance and pipette capacitance neutralization adjustment throughout the experiment. Recordings were made from −60 mV to approximately resemble the resting membrane potential of bushy cells (McGinley and Oertel, 2006; Price and Trussell, 2006; Milenković et al., 2007). Voltages were corrected off-line by subtracting empirically determined junction potentials of 14 mV from holding potential (V_hold_; Neher, 1992). Recorded signals were low-pass filtered at 5 kHz and sampled at 20 kHz. Data analysis was done with pClamp 10 software (Molecular Devices) and Matlab based scripts.

The half-width of APs (APhw), elicited by a 200 ms depolarizing current injection, was analyzed at the half-maximal AP amplitude between the AP threshold and the AP peak. The AP threshold was determined as the maximum of the second derivative preceding AP. For each cell, the analysis was based on averaged data from >10 repetitions of a suprathreshold current injection eliciting at least one AP.

#### Morphological Characterization of Recorded Neurons

*Post hoc* labeling of biocytin-filled neurons was used do distinguish BCs and SCs according to their specific morphology (Milenkovic et al., 2009; Dietz et al., 2012). After recording, the slices were fixed overnight in 4% paraformaldehyde [PFA in 0.1 M phosphate-buffered saline (PBS), pH 7.3]. Then, the slices were washed (6 × 5 min with TBS and 6 × 5 min with TBS / 0.3% Triton X-100) and incubated with Cy2-conjugated streptavidin (5 μg/ml, Jackson Immunoresearch Lab, West Grove, PA, USA) for 2.5 h at RT. Images were generated with a confocal laser scanning microscope (TCS SP5, Leica).

### Statistics

Statistical analysis was performed with Matlab and SigmaPlot (version 10, Systat, RRID:SCR_003210). Data sets were tested for Gaussian distribution prior to comparison by analysis of variance (ANOVA) followed by pairwise multiple comparisons (Holm-Šidák *post hoc* test). One-way ANOVA was used when comparing the effects of age within one cell type, and two-way ANOVA when comparing the effects of age within both BCs and SCs. One-way repeated measurements ANOVA has been employed to compare the effects of different stimuli in the same cells. In box plots, dots representing single cells are added to show data distribution. Average data are reported as mean ± SD or median with 25 and 75 percentiles, depending on the distribution.

## Results

The development of AP firing properties was investigated in the two principal neuron types of the AVCN, BCs and SCs. Juxtacellular *in vivo* recordings were acquired from anesthetized mice starting at hearing onset (P12), throughout the early post-hearing period (P13–P18) up to young adulthood (P30). The onset of hearing in mice was reported to occur between P9–14 (Alford and Ruben, 1963; Hack, 1968; Ehret, 1976; Sonntag et al., 2009). In our colony, the external auditory meatus typically opened between P11 and P12. Recordings in two P11 mice revealed acoustically evoked neuronal responses in only 2/8 cells. Hearing thresholds in these two units were 80 and 85 dB SPL, while in the remaining six units, auditory stimuli up to 90 dB SPL had no impact on discharge rate. In mice P12 and older, all units responded to acoustic stimulation. AP firing was recorded in 136 units in the absence of acoustic simulation (spontaneous activity) and during the presentation of pure tones and SAM tones. In mice aged P18 and P30, the two principal AVCN neuron types could unequivocally be classified as BCs or SCs, based on the presence or absence of prepotentials, respectively. Prepotentials indicate presynaptic AP at the endbulb of Held terminal preceding the postsynaptic AP of BCs. Units with prepotentials show primary-like discharge patterns in response to pure tone stimulation, while SCs lack prepotentials and display chopper response patterns (Young et al., 1988; Blackburn and Sachs, 1989; Typlt et al., 2012). Shortly after hearing onset (P12–14), the respective criteria did not allow for a clear cell type differentiation because some units lacked a noticeable prepotential or a chopper PSTH.

However, slice recordings revealed longer AP duration in BCs compared to SCs (cat: Bal and Baydas, 2009; dog: Bal et al., 2009; mouse: Yang et al., 2016). To test whether the APs differ between fairly immature BCs and SCs, current clamp recordings were conducted in acute slices from P10–18 mice. APs were evoked with depolarizing current injections of increasing amplitudes until the threshold was reached, i.e., ≥1 AP was elicited. BCs generated mostly a single or at most few APs, while SCs fired at least 13 APs during a 200 ms current pulse (Figures 1A,B). The electrophysiological characterization, consistent with type II and type I discharge patterns of AVCN neurons (Oertel, 1983; Wu and Oertel, 1984; Francis and Manis, 2000), was confirmed by biocytin labeling revealing BC and SC morphology, respectively (Oertel et al., 1990; Cao et al., 2007; Lauer et al., 2013; Xie and Manis, 2017). Generally, the AP half width was smaller in SCs compared to BC (Figure 1C; *p* = 0.015, two-way ANOVA). In BCs, the AP half width decreased by 65% from 0.94 ± 0.06 ms at P10–11 to 0.33 ± 0.03 ms at P14 (*p* < 0.001, two-way ANOVA). In SCs, the AP half width shortened by 74% from 0.73 ± 0.15 ms at P10–11 to 0.19 ± 0.004 ms at P14 (*p* < 0.001, two-way ANOVA). Between P14 and P18, there was no further AP shortening in neither of the cell types (BCs *p* = 0.78; SCs *p* = 0.8).

Based on these results, we hypothesized that the AP kinetics could be used as an additional criterion to distinguish between immature BCs and SCs recorded *in vivo* (Figure 1D). To test this, we quantified the AP repolarization slopes calculated as the normalized voltage change between 95% and 5% of the AP falling phase (Figure 1E). The hierarchical clustering was employed to the data to separate distinct populations of units. The results were evaluated using silhouette values for each age group. Silhouette values are constrained between [−1, 1], with higher values indicating well-matched objects within a cluster. At ages P18 and P30, the units clustered in two separate populations [median silhouette with 25 and 75 percentiles for P18 = 0.91 (0.76; 0.97), *n* = 28; P30 = 0.93 (0.9; 0.95), *n* = 27]. Units with shallower repolarization slope (P18: 1.8–3, *n* = 16; P30: 1.5–4.1, *n* = 14) showed primary-like PSTH (CV_PSTH_ > 0.4), and prominent prepotentials, the hallmarks of BCs. Units with steeper repolarization slopes (P18: 3.6–6, *n* = 12; P30: 4.3–5.6, *n* = 13) displayed chopper PSTH (CV_PSTH_ < 0.4) characteristic for SCs. Accordingly, the less mature units from P12–14 that exhibited a prepotential had smaller slope values (red symbols for P12–14 Figure 1E), and chopper units had higher slope values (blue symbols for P12–14 Figure 1E). The remaining P12–14 units exhibiting neither a prepotential nor a chopping pattern clustered well within these two cell populations [*black symbols*; median silhouette with 25 and 75 percentiles P12 = 0.92 (0.83; 0.94), *n* = 22; P13 = 0.91 (0.86; 0.96), *n* = 33; P14 = 0.89 (0.78; 0.94), *n* = 26]. In conclusion, our data strongly support earlier studies suggesting different AP duration between BCs and SCs, which can be used for presumptive classification of immature units, despite the ambiguity of temporal response patterns. These classification results were used for* de facto* identification in the remainder of the study.

### Prolonged Maturation of Intrinsic Excitability

The properties of APs in auditory brainstem neurons are determined by specific subsets of sodium and potassium channels and their precise cellular localization (Trussell, 1999; Kuba and Ohmori, 2009; Johnston et al., 2010; Brown and Kaczmarek, 2011; Hong et al., 2018). The resulting timing accuracy of APs is decisive for binaural auditory processing (Oertel, 1999; Grothe and Klump, 2000; Kopp-Scheinpflug and Forsythe, 2018). The developmental changes in BC- and SC-AP characteristics were investigated by analyzing signal kinetics during spontaneous activity. In 76% (53/70) of units classified as BC the signals displayed a prepotential followed by a postsynaptic AP (Figure 1F, left). All units classified as SCs had a biphasic waveform, with a steep monotonic depolarization and similarly steep repolarization (Figure 1F, right). The signal waveform in juxtacellular recordings approximately corresponds to the first derivative of the intracellularly recorded AP (Lorteije et al., 2009). Presently, the AP duration was approximated as the time between the signal maximum and minimum (i.e., time from positive to negative peak). In the course of development, APs became shorter in both cell types. Yet, APs in SCs were consistently shorter than in BCs (Figure 1G; Table 1; *p* < 0.001, two-way ANOVA). In BCs, the AP duration decreased by 30% from 0.64 ms [0.61; 0.87] at P12 to 0.45 ms [0.38; 0.56] at P30 (*p* < 0.001, two-way ANOVA). In SCs, the APs shortened by 37% from 0.38 ms [0.34; 0.41] at P12 to 0.24 ms [0.23; 0.28] at P30 (*p* < 0.001, two-way ANOVA). The maturation of sodium and potassium conductance, which tightly regulate AP properties of time-coding auditory brainstem neurons (Manis and Marx, 1991; Brew and Forsythe, 1995; Scott et al., 2005; Klug and Trussell, 2006; Cao et al., 2007; Yang et al., 2016), probably accounts for the developmental shortening of BC- and SC-APs. In conclusion, these data confirm observations from slice experiments and suggest that differences in AP duration can be used as a parameter to separate BCs and SCs in *in vivo* extracellular recordings.

**Table 1:** Functional properties of bushy cells (BCs) and stellate cells (SCs) during development.

Parameter	Cell type	P12	P13	P14	P18	P30
Sample size	BC	10	17	13	16	14
	SC	12	16	13	12	13
AP duration (ms)	BC	0.64 [0.61; 0.87]	0.59 [0.53; 0.64]	0.53 [0.5; 0.6]	0.48 [0.42; 0.53]	0.45 [0.38; 0.56]
	SC	0.38 [0.34; 0.41]	0.32 [0.28; 0.36]	0.31 [0.26; 0.32]	0.29 [0.24; 0.32]	0.24 [0.23; 0.28]
PP-AP delay (ms)	BC	0.93 ± 0.1 *n = 5	0.9 ± 0.11 *n = 9	0.72 ± 0.08 *n = 9	0.75 ± 0.07	0.63 ± 0.07
Spontaneous firing rate (AP/s)	BC	22.2 [7; 41.5]	31.4 [1.8; 41.2]	43.9 [3.9; 68.2]	61.8 [26.1; 84.4]	50.8 [9.4; 85.7]
	SC	1.6 [0.7; 7.7]	3.6 [1.1; 16.2]	6.6 [0.9; 12.1]	17.4 [6.6; 42.1]	33.6 [9.7; 58.9]
CV_spont_	BC	1.14 [0.97; 1.41]	1.01 [0.93; 1.11]	0.8 [0.72; 0.94]	0.78 [0.7; 0.9]	0.9 [0.76; 0.98]
	SC	1.56 [1.2; 1.82]	1.24 [1.08; 1.6]	1.12 [0.98; 1.42]	0.92 [0.86; 1]	0.91 [0.83; 1.06]
No of bursts per 100 s	BC	0.55 [0; 1.7]	0 [0; 0]	0 [0; 0]	0 [0; 0]	0 [0; 0]
	SC	1.45 [0.35; 3]	0.65 [0; 2.6]	0 [0; 2.76]	0 [0; 0]	0 [0; 0]
Threshold (dB SPL)	BC	44.6 [39.2; 53.4]	23.7 [16.3; 31.8]	11.8 [−0.8; 21.3]	5.6 [0.2; 15.2]	4 [−2; 7.7]
	SC	43.4 [37; 46.1]	26.1 [19.2; 31.6]	7 [−4.2; 11.6]	8.2 [2.9; 19.7]	−0.4 [−4; 2.8]
Maximum firing rate (AP/s)	BC	171.2 ± 48.8	204.1 ± 52.6	228.2 ± 53	262.2 ± 53	317.5 ± 60.1
	SC	169.8 ± 53.1	203.2 ± 51.8	275.6 ± 75.8	334 ± 76	438.3 ± 105.8
Dynamic range (dB SPL)	BC	14.1 ± 5.4	21.9 ± 6.8	25 ± 10	32.4 ± 13.9	29.7 ± 15.8
	SC	17.6 ± 6.2	19.1 ± 8.9	21.9 ± 9.6	24.4 ± 13.7	28.9 ± 14.4
Inhibition strength (% of spont rate)	BC	–	–	61.1 [42; 80.6] *n = 7	57.7 [48.4; 70.4] *n = 7	67.1 [61.8; 76.8] *n = 12
	SC	–	96.9 [89.6; 100] *n = 8	100 [94.8; 100] *n = 6	94.8 [89; 100] *n = 8	98 [93.5; 100] *n = 10
Q_20_inh	BC	–	–	21.7 [14.3; 31.1] *n = 7	8.6 [3.9; 17.8] *n = 7	12.9 [5.6; 29.2] *n = 12
	SC	–	5.8 [4.7; 7.3] *n = 8	6.3 [4.3; 21.2] *n = 6	6.2 [2.9; 15.4] *n = 8	8.8 [3.8; 10.4] *n = 10
CV_PSTH_	BC	0.48 [0.46; 0.55]	0.46 [0.44; 0.49]	0.46 [0.44; 0.49]	0.53 [0.48; 0.56]	0.5 [0.46; 0.54]
	SC	0.48 [0.42; 0.54]	0.39 [0.28; 0.52]	0.47 [0.43; 0.52]	0.21 [0.18; 0.27]	0.33 [0.16; 0.37]
First spike latency (ms)	BC	6.2 ± 0.8	6.5 ± 0.8	4.8 ± 0.7	4.3 ± 0.4	4.1 ± 0.5
	SC	8.3 ± 0.8	7.2 ± 1.3	6 ± 0.4	5.9 ± 0.3	5.8 ± 0.7

*Most parameters were examined in all recorded cells, indicated by sample size in the first row. Deviating sample sizes are marked with an asterisk. Data are presented as mean ± SD or as median with 25 and 75 percentiles, depending on the distribution*.

To assess the developmental changes of the transmission delay, we took advantage of the BC’s complex signal waveform, exhibiting the prepotential of the endbulb of Held (Typlt et al., 2010). The shortening of the prepotential-AP time continued well after hearing onset, with significant reduction between P13 and P14 and between P18 and P30 (Figure 1H; Table 1; *p* < 0.001, one-way ANOVA). This could be potentially caused by the maturation of pre- and postsynaptic components.

### Delayed Maturation of Spontaneous Firing in SCs

Before hearing onset, immature IHCs generate bursting activity in ANFs, which is conveyed along the afferent auditory pathway (Jones et al., 2007; Sonntag et al., 2009; Tritsch et al., 2010). In addition to the auditory nerve input, the intrinsically driven I_h_-current contributes to spontaneous firing of the cochlear nucleus neurons (Yin et al., 2018). The patterned activity changes into Poisson-like firing around hearing onset (Sonntag et al., 2009; Crins et al., 2011) and mature cochlear nucleus neurons exhibit Poisson-like firing in the absence of sound (Rodieck et al., 1962; Pfeiffer and Kiang, 1965; Kopp-Scheinpflug et al., 2008).

The presently recorded spontaneous rates in BCs and SCs increased during the early auditory experience, with consistently higher firing in BCs compared to SCs (Figures 2A,B; Table 1; *p* < 0.001, two-way ANOVA). The rates were highly variable between the cells, similar to recordings from ANF in adult CBA mice (Taberner and Liberman, 2005). The maximal spontaneous rate was 152 AP/s in a P30 BC, and 131 AP/s in a P18 SC. At all ages, a notable fraction of BCs and SCs had spontaneous firing rates below 18 AP/s (P12–P14: BCs 40% and SCs 88%; P18 and P30: BCs 27% and SCs 44%). Hence, it is conceivable that the respective units were innervated by low and medium spontaneous ANFs (Liberman, 1978, 1991; Ryugo and Rouiller, 1988), but it cannot be excluded that still immature biophysical and synaptic properties contributed to low firing.

At the hearing onset, the firing in BCs and SCs displayed transitional patterns between bursting and Poisson-like (Figure 2A). Thereafter, a gradual decrease of the coefficient of variation (CV_spont_) indicated the reduction of patterned activity (Figure 2C; Table 1; *p* < 0.001, two-way ANOVA). Notably, the developmental cessation of bursting was faster in BCs than in SCs (Figure 2D). While 46% (6/13) of SCs still showed bursts at P14, BCs had no longer bursting pattern (Figure 2D; Table 1; *p* = 0.002, two-way ANOVA). The duration of bursts generally decreased between P12 and P13 (*p* = 0.04, two-way ANOVA), showing no difference between the cell types (Figure 2E). Considering that both neuron types are driven by ANF inputs, the prolonged bursting in SCs may be caused by the delayed development of synaptic inputs and of intrinsic excitability.

### Rapid Development of Hearing Sensitivity and Hearing Range

**Figure 3:**
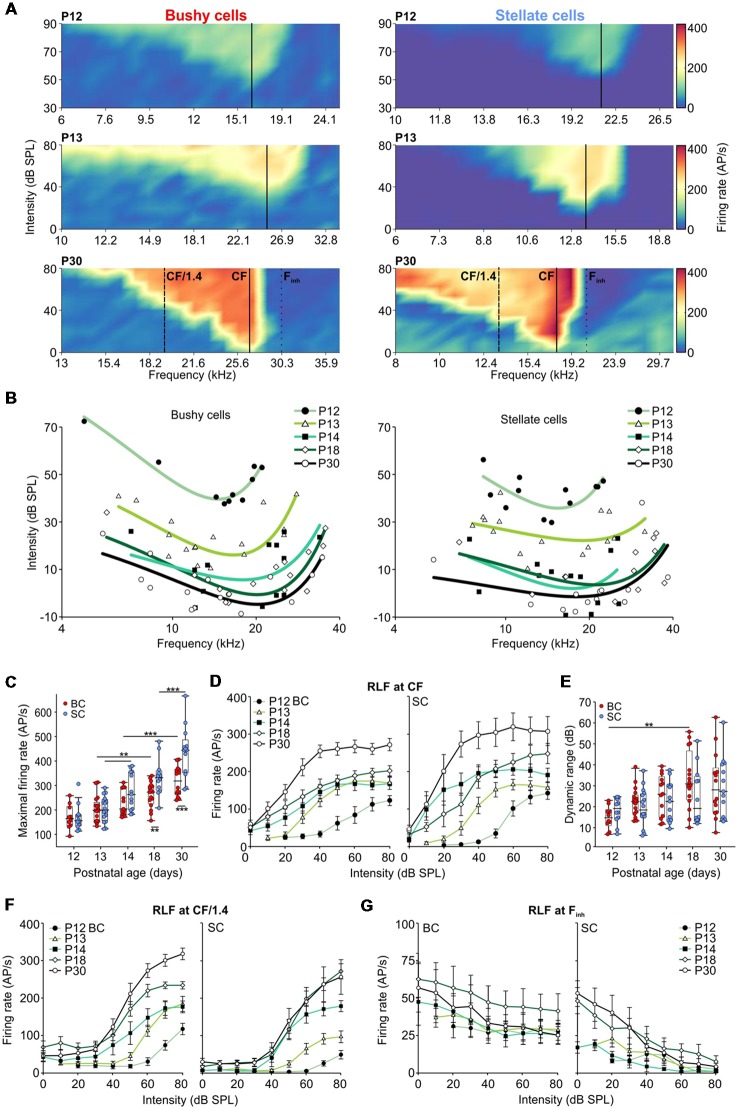
Development of hearing threshold and frequency response area in AVCN units. **(A)** Representative frequency response areas in BCs (left), and SCs (right) at P12, P13, and P30 (upper, middle and lower panels, respectively). For better comparison, the color scaling is identical for all frequency response areas indicating spike discharge rates between 0 (dark blue) and 420 APs per second (red). Solid black lines indicate the units’ characteristic frequencies (CFs). Note the general increase in firing rates within the excitatory response area. SCs show a stronger reduction in firing activity towards the low-frequency tail compared to BCs. Solid lines (CF), dashed lines (half octave below CF [CF/1.4]), and dotted lines (high-frequency inhibitory sideband [F_inh_]) depict the positions where the respective rate level functions were calculated. **(B)** Trend curves based on CF and threshold values of BCs (left) and SCs (right). For each age group, a polynomial-quadratic fit of the threshold values was used to approximate hearing range and sensitivity. Both cell types follow similar maturational pattern exhibiting decreasing response thresholds (effect of cell type *p* = 0.35, effect of age *p* < 0.001, interaction cell type × age *p* = 0.52; two-way ANOVA), and the development of high-frequency hearing between P12 and P14. **(C)** Maximum firing rates during acoustic stimulation increased from P12 to P30 in both BCs (red) and SCs (blue; effect of cell type *p* < 0.001, effect of age *p* < 0.001, interaction cell type × age *p* = 0.014, two-way ANOVA). Box-plots show medians, the 25 and 75 percentiles, and the interdecile ranges (**p < 0.01; ***p < 0.001). **(D)** Average rate level functions for P12 to P30 calculated at a CF. **(E)** The dynamic ranges of rate level functions at CF expand similarly during development for both, BCs and SCs (effect of cell type *p* = 0.36, effect of age *p* = 0.005, interaction cell type × age *p* = 0.26; two-way ANOVA). **(F)** Average rate level functions obtained at half an octave below CF show bigger dynamic ranges in BCs (effect of cell type *p* < 0.001, effect of age *p* = 0.02, interaction cell type × age *p* = 0.92; two-way ANOVA) and lower firing rates in SCs up to the stimulation level of 60 dB SPL (10–30 dB SPL: effects of cell type *p* < 0.001, effects of age *p* < 0.001, interaction cell type × age at 10 dB *p* = 0.61, at 20 dB *p* = 0.3, at 30 dB *p* = 0.71; 40 and 50 dB SPL: effects of cell type *p* < 0.01, effects of age *p* < 0.001, interaction cell type × age at 40 dB *p* = 0.82, at 50 dB *p* = 0.22; 60 dB SPL: effect of cell type *p* = 0.02, effect of age *p* < 0.001, interaction cell type × age 0.76; two-way ANOVA). **(G)** The average rate level functions at the F_inh_. Note the stronger effect of inhibition in SCs compared to BCs (10 dB SPL: *p* = 0.009; 20 dB SPL: *p* = 0.003; 30–80 dB SPL: *p* < 0.001, two-way ANOVA).

Neurons of the AVCN receive primary excitatory input through ANFs (Brawer and Morest, 1975; Schwartz and Gulley, 1978; Nicol and Walmsley, 2002), and prominent inhibitory inputs originating in the CN (Wickesberg and Oertel, 1990; Saint Marie et al., 1991; Campagnola and Manis, 2014) and in superior olivary complex (Benson and Potashner, 1990; Ostapoff et al., 1990, 1997; Schofield, 1991; Warr and Beck, 1996). Integration of both excitatory and inhibitory inputs defines the neuronal response properties to acoustic stimulation. The functional development of respective inputs can be explored by quantifying the units’ excitatory and inhibitory receptive fields. Excitatory responses were tested by presenting pure tone pulses with 200 frequency/intensity combinations and measuring the units’ frequency response areas. Both BCs and SCs exhibited characteristic V-shaped excitatory frequency response areas with response thresholds rapidly decreasing after the onset of hearing (Figures 3A,B; *p* < 0.001, two-way ANOVA). The developmental time course was comparable in both cell types with adult-like thresholds gained by P14 (Figures 3A,B; Table 1; BC: 3.8 ± 12.8 dB SPL; SC: −2.1 ± 11 dB SPL). During this early phase of development, the CF range covered by the units gradually extended towards high frequencies (Figure 3B), consistent with earlier ABR recordings in mice (Song et al., 2006). At P12, characteristic frequencies were limited between 4.8 and 22.4 kHz, while at P14 the high-frequency range extended to 33.9 kHz. Due to technical limitations of the sound delivery system, the maximum sound frequency tested in this study was 50 kHz. At P30, the highest CF for a single-unit recording was 38.2 kHz and for a multiunit recording 47 kHz.

Both BCs and SCs showed a continuous increase in maximal acoustically evoked discharge rates from P12 to P30 (Figure 3C; Table 1; *p* < 0.001, two-way ANOVA). Up to P14 the developmental courses were comparable in both cell types, but thereafter the rate increase in SCs surpassed that of BCs (BC vs. SC: P18: *p* = 0.006; P30: *p* < 0.001; two-way ANOVA). Altogether, both BCs and SCs exhibited more than a two-fold increase in maximal firing rates between P12 and P30. Several mechanisms may account for this change, such as the increase in firing rates of ANFs, maturation of the transmitter release machinery and/or changes in excitability of the postsynaptic neurons.

### Rate-Level Functions Reveal Stronger Inhibitory Effects in SCs

RLFs were calculated from the frequency response area across units’ excitatory and inhibitory receptive fields, i.e., at the unit’s CF, at half-octave below CF and within the “high-frequency inhibitory sideband” (F_inh_). Average rate level functions at the units’ CF had a similar developmental profile for BCs and SCs showing a gradual increase up to P30 (Figure 3D). Also, the dynamic range (DynR) increased during maturation in both cell types (Figure 3E; Table 1; *p* = 0.005, two-way ANOVA). There was no correlation between the dynamic range and CF in either of the cell types (BC: *p* = 0.93, *n* = 70; SC: *p* = 0.39, *n* = 66; Spearman’s rank correlation).

Comparison of rate level functions at half-octave below CF (“CF/1.4”, Figure 3F) showed a developmental increase in firing rates in both cell types, but the rates in SCs were consistently smaller than in BCs (for 10, 20 and 30 dB SPL *p* < 0.001, for 40 and 50 dB SPL *p* < 0.01, for 60 dB *p* = 0.02; two-way ANOVA). Due to the prominent inhibition at the low-frequency flank of the frequency response area, SCs had a smaller dynamic range at CF/1.4 with respect to BCs across all age groups (*p* < 0.001, two-way ANOVA).

Inhibitory sidebands at frequencies above CF were described earlier in AVCN units of the cat (Rhode and Greenberg, 1994b), guinea pig (Winter and Palmer, 1990), rat (Paolini et al., 2005), and gerbil (Typlt et al., 2012; Nerlich et al., 2014b; Keine and Rübsamen, 2015). Among BCs with sufficiently high spontaneous activity, allowing for quantification of inhibition (SR > 8 AP/s), 1/7 at P12 and 2/9 at P13 exhibited inhibitory sidebands. In SCs, 2/3 showed an effect of inhibition already at P12, and at P13 8/8 underwent a complete block of activity when stimulated within the (F_inh_) at SPLs >40 dB. The strength of inhibition was quantified as the relative reduction of spontaneous firing evoked at stimulation 20 dB above the threshold at F_inh_ (Nerlich et al., 2014b; Keine and Rübsamen, 2015). Stimulation at F_inh_ more potently reduced AP firing in SCs than in BCs (Figure 3G; Table 1; effect of cell type *p* < 0.001, effect of age *p* = 0.44, interaction cell type × age *p* = 0.78; two-way ANOVA). The bandwidth of the inhibitory sideband was measured 20 dB above the inhibitory threshold and expressed as Q_20_ values. This analysis revealed a broader frequency range (smaller Q_20_) of inhibitory sidebands in SCs (Table 1; effect of cell type *p* = 0.03, effect of age *p* = 0.26, interaction cell type × age *p* = 0.63; two-way ANOVA). Together, these findings suggest a stronger and spectrally broader acoustically evoked inhibition on SCs compared to BCs.

### Delayed Development of Temporal Response Properties in SCs

**Figure 4:**
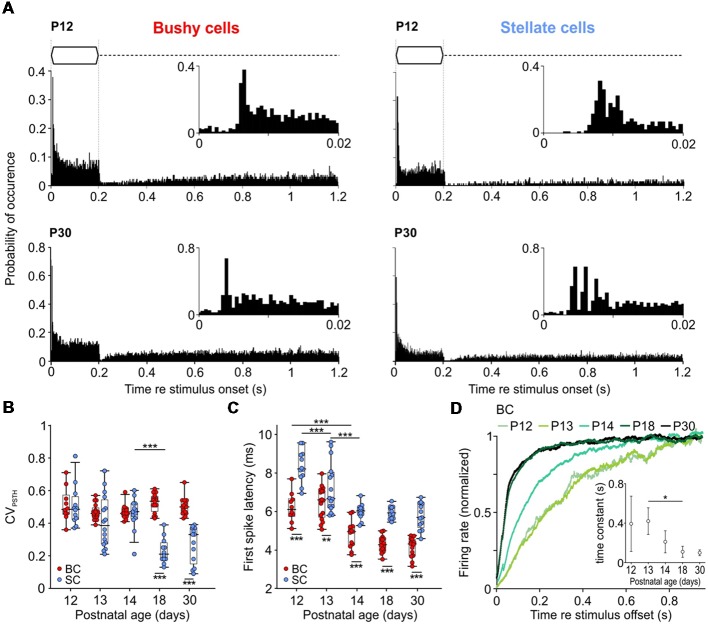
Development of temporal response properties to tone burst stimulation. **(A)** Peristimulus time histograms (PSTHs) of representative BCs and SCs at P12 (top) and P30 (bottom); bin width 0.5 ms. Insets show an enlargement of the first 20 ms of respective responses. Note the characteristic primary-like pattern of BC (bottom left) and chopping pattern of SC (bottom right) at P30. At P12 (upper panels), response latencies are longer in both cell types and SCs lacks chopping discharge pattern. **(B)** BCs and SCs show comparable CV_PSTH_ values at P12–14 which diverge from P18 on (effect of cell type *p* < 0.001, effect of age *p* < 0.001, interaction cell type × age *p* < 0.001; two-way ANOVA). Dots indicate values of individual units; box-plots show medians, 25*th* and 75*th* percentiles, and the interdecile ranges. **(C)** First spike latency (FSL) decreases with age in both cells types, with overall lower values in BCs (effect of cell type *p* < 0.001, effect of age *p* < 0.001, interaction cell type × age *p* = 0.009; two-way ANOVA). **(D)** Recovery of spontaneous discharge activity after stimulus offset in BCs. Each trace shows averaged data for 6–10 cells. Spontaneous firing for each cell was calculated as the mean of 150 post-sound stimulation periods. Inset shows time constant values calculated from the fit (single exponential for P12 and P13, bi-exponential for P18 and P30) to the averaged data for each age group (mean ± standard deviation (SD), *p* < 0.001; one-way ANOVA) [*p < 0.05, **p < 0.01; ***p < 0.001].

Temporal response properties of BCs and SCs were assessed by analyzing PSTHs computed from 200 repetitions of pure-tone stimulation at the units’ CF (Figure 4A). Consistent with earlier studies (Young et al., 1988; Blackburn and Sachs, 1989), BCs showed a primary-like PSTH, characterized by a phasic onset component followed by sustained activity throughout the duration of the stimulus (Figure 4A, left). In contrast, SCs showed a characteristic chopper PSTH in animals >P18, defined by a regular sequence of APs triggered at the stimulus onset, resulting in low CV_PSTH_ values (Rhode and Smith, 1986; Young et al., 1988; Typlt et al., 2012). However, in young animals, the chopper PSTH was present only in a subset of SCs (40% and 38% at P12 and P13–P14, respectively). At P18 and P30, all mice showed an initial chopping firing pattern which transitioned into random sustained firing activity (Figure 4A, bottom right). These cells had lower CV_PSTH_ values compared to P12–P14 SCs, and to age-matched BCs (Figure 4B; Table 1; *p* < 0.001, two-way ANOVA). Several factors contribute to the adult-like chopper discharge pattern, including high-voltage activated potassium conductance and the temporally precise activity of excitatory and inhibitory inputs (Trussell, 2002; Oertel et al., 2011). The lack of characteristic chopper PSTH in animals <P18 might indicate that synaptic inputs onto SCs still mature during the third postnatal week.

The PSTHs were further used to measure the delay of AP firing (FSL) upon pure tone stimulation. Both cell types showed mature-like FSL already at P14 (Figure 4C). However, BCs had consistently shorter FSL than SCs (Table 1; *p* < 0.001, two-way ANOVA).

Following the offset of acoustic stimulation, the firing in AVCN units is transiently reduced below the spontaneous activity level (Kopp-Scheinpflug et al., 2002). The dynamics of recovery from AP depression was quantified only in BCs by fitting an exponential function to the recovery time course (Figure 4D). The generally low spontaneous rates in SCs did not allow for a respective analysis. The recovery time constants were comparable at P12 and P13 BCs (P12 = 407.6 ± 253.4 ms, *n* = 6; P13 = 420.3 ± 135.3 ms, *n* = 5), and became progressively shorter thereafter to reach adult-like values by P18 (P14 = 210.6 ± 111.9 ms, *n* = 10; P18 = 109.5 ± 55.9 ms, *n* = 6; P30 = 99.3 ± 29.1 ms, *n* = 5; *p* < 0.001; one-way ANOVA). Notably, the time course was best fitted with mono-exponential function for P12–P14, while P18 and P30 animals showed bi-exponential dynamics of recovery (P18: τ_fast_ = 126.7 ± 70.4 ms, τ_slow_ = 322.8 ± 206.8 ms; P30: τ_fast_ = 112.7 ± 45.8 ms, τ_slow_ = 303.4 ± 131.6 ms).

The present data suggest that in P18 and P30 BCs two factors might contribute to the fast recovery of spontaneous activity after the offset of acoustically evoked activity: the then established maturity of the cochlea and of the endbulb of Held synapses.

### Similar Developmental Time Course, but Better SAM Processing in Mature-Like BCs Than in SCs

**Figure 5:**
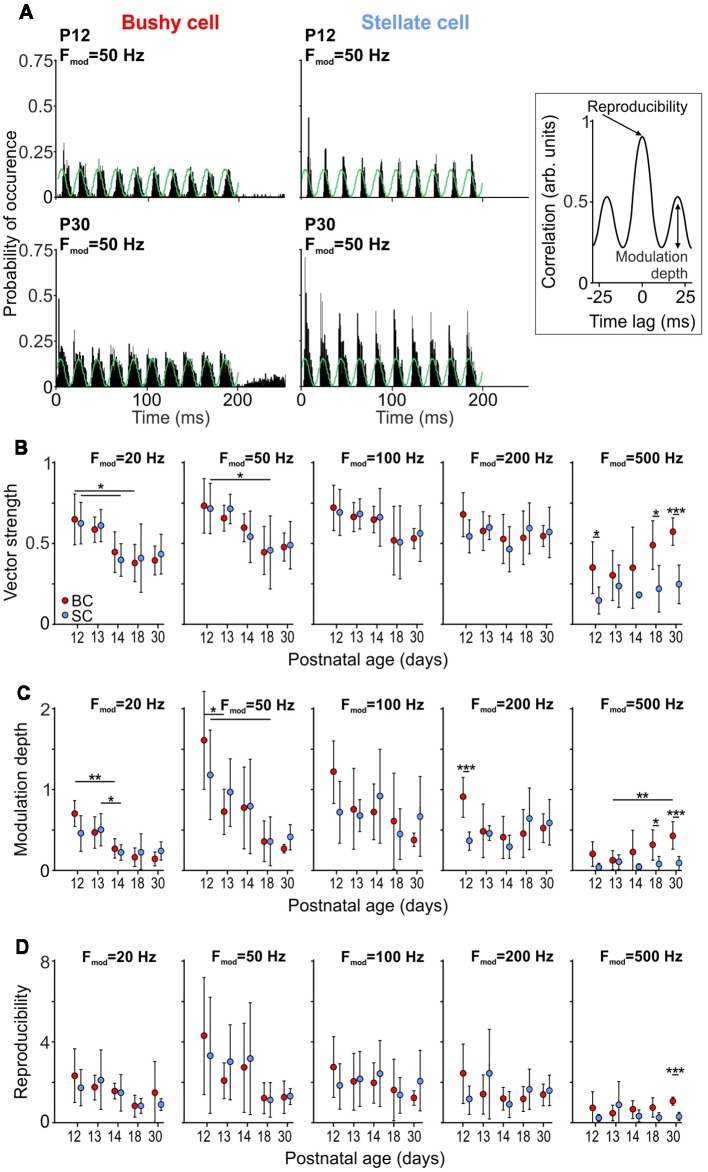
Development of responses to sinusoidal amplitude modulated (SAM) tone bursts. **(A)** PSTHs of responses to 150 repetitions of SAM stimulation at 50 Hz modulation frequency for BCs (left) and SCs (right) at P12 (top) and P30 (bottom). Note the increased firing rates in both cells types at P30. Right: schematic of a cross-correlation function used to calculate modulation depth **(C)** and reproducibility **(D)** between trials. Modulation depth was defined as the SD of the first cycle and the reproducibility as the peak of the normalized cross-correlation. **(B)** The vector strength (VS) at *F*_mod_ = 20 and 50 Hz decreases with maturity for both BCs and SCs (*F*_mod_ = 20 Hz; effect of cell type *p* = 0.91, effect of age *p* < 0.001, interaction cell type × age *p* = 0.9; *F*_mod_ = 50 Hz; effect of cell type *p* = 0.93, effect of age *p* < 0.001, interaction cell type × age *p* = 0.91; two-way ANOVA), while showing no age-dependence for *F*_mod_ = 100–500 Hz (*F*_mod_ = 100 Hz: effect of cell type *p* = 0.9, effect of age *p* = 0.1, interaction cell type × age *p* = 0.99; *F*_mod_ = 200 Hz: effect of cell type *p* = 0.57, effect of age *p* = 0.3, interaction cell type × age *p* = 0.35; two-way ANOVA). Symbols indicate mean ± SD. For *F*_mod_ = 500 Hz, the VS is generally higher in BCs than in SCs (effect of cell type *p* < 0.001, effect of age *p* = 0.03, interaction cell type × age *p* = 0.19; two-way ANOVA). **(C)** In both cell types, modulation depth at *F*_mod_ = 20 and 50 Hz decrease with maturity (*F*_mod_ = 20 Hz; effect of cell type *p* = 0.65, effect of age *p* < 0.001, interaction cell type × age *p* = 0.13; *F*_mod_ = 50 Hz; effect of cell type *p* = 0.89, effect of age *p* < 0.001, interaction cell type × age *p* = 0.45; two-way ANOVA). There is no age-dependent effect for *F*_mod_ 100–500 Hz (*F*_mod_ = 100 Hz: effect of cell type *p* = 0.67, effect of age *p* = 0.11, interaction cell type × age *p* = 0.25; *F*_mod_ = 200 Hz: effect of cell type *p* = 0.21, effect of age *p* = 0.12, interaction cell type × age *p* = 0.02; two-way ANOVA). Comparison between the cell types shows larger modulation depth in BCs for *F*_mod_ = 500 Hz (*F*_mod_ = 500 Hz: effect of cell type *p* < 0.001, effect of age *p* = 0.03, interaction cell type × age *p* = 0.05; two-way ANOVA). D, Reproducibility shows similar developmental pattern for BCs and SCs, being reduced with age at *F*_mod_ = 20 and 50 Hz (*F*_mod_ = 20 Hz; effect of cell type *p* = 0.44, effect of age *p* = 0.02, interaction cell type × age *p* = 0.65; *F*_mod_ = 50 Hz; effect of cell type *p* = 0.88, effect of age *p* = 0.01, interaction cell type × age *p* = 0.78; two-way ANOVA), and without changes at *F*_mod_ = 100–500 Hz (*F*_mod_ = 100 Hz: effect of cell type *p* = 0.93, effect of age *p* = 0.59, interaction cell type × age *p* = 0.58; *F*_mod_ = 200 Hz: effect of cell type *p* = 0.96, effect of age *p* = 0.46, interaction cell type × age *p* = 0.13; *F*_mod_ = 500 Hz: effect of cell type *p* = 0.03, effect of age *p* = 0.83, interaction cell type × age *p* = 0.1; two-way ANOVA) [*p < 0.05; **p < 0.01; ***p < 0.001].

In a natural context, most behaviorally relevant acoustic stimuli feature modulations in sound amplitude (Attias and Schreiner, 1998; Varnet et al., 2017). The neuronal encoding of stimulus envelope can be experimentally assessed using SAM sounds (Moller, 1974; Palmer, 1982; Frisina et al., 1990; Joris and Yin, 1992). To investigate how SAM coding develops in BCs and SCs, tones were presented at the unit’s CF, 20 dB SPL above threshold with an amplitude modulation at 20 Hz, 50 Hz, 100 Hz, 200 Hz, and 500 Hz (*F*_mod_, Figure 5). Coupling of the neuronal discharges to the phase of the modulation cycle was quantified by VS, which provides an estimate of the temporal precision in spike responses to the amplitude envelope (Figure 5B). The VS values at *F*_mod_ = 20 Hz and *F*_mod_ = 50 Hz were highest at P12 and P13 for both BCs and SCs, implying decreased temporal precision with maturity (*p* < 0.001, two-way ANOVA). However, this seemingly paradoxical result can be explained by the lower response thresholds in older animals, and thus higher number of APs generated per cycle at comparable above-threshold values (see P12 and P30 in Figure 3A). At *F*_mod_ 100 Hz and 200 Hz both cell types had similar VS across ages, suggesting that synchronization of firing to the stimulus envelope develops early. Unlike for *F*_mod_ ≤ 200 Hz, the temporal coupling to *F*_mod_ = 500 Hz was higher in BCs (*p* < 0.001, two-way ANOVA). VS increased with *F*_mod_ for P18 and P30 BCs, consistent with an improvement of phase coupling (P18: *p* = 0.004; P30: *p* = 0.009; one-way RM ANOVA). SCs of the same age also showed improved phase coupling for *F*_mod_ ≤ 200 Hz (P18: *p* < 0.001; P30: *p* < 0.001; one-way RM ANOVA). However, the phase coupling in SCs deteriorated at *F*_mod_ = 500 Hz, resulting in low VS values. This result is consistent with a drop in VS of chopper units at modulation frequencies around 500 Hz, observed earlier in guinea pigs (Sayles et al., 2013).

While VS measures spike synchrony to the phase of amplitude modulation, it provides only limited information about the firing rate modulation by the SAM stimulus, and high VS values cannot *per se* be equated with the faithful encoding of stimulus envelopes. We, therefore, calculated the normalized within-cell across-trial cross-correlation, as described earlier (Joris et al., 2006; Keine et al., 2016). The firing rate modulation to the SAM stimulus was quantified as the SD of the first cycle of the cross-correlogram, which is a measure of firing rate modulation depth (inset Figures 5A,C). A strong firing modulation evoked by SAM stimuli will result in high SD values of the cross-correlogram, consistent with high values for modulation depth. If, on the contrary, the neuronal response was not modulated by the SAM stimulus, the SD of the cross-correlogram and the modulation depth would be close to zero. For *F*_mod_ = 20 Hz and 50 Hz, both cell types showed a considerable decrease of modulation depth with age (*p* < 0.001, two-way ANOVA). In contrast, the modulation depth at *F*_mod_ = 100 Hz and 200 Hz was similar throughout the developmental period investigated. Notably, at P18 and P30, BCs showed a higher modulation depth than SCs for *F*_mod_ = 500 Hz (*p* < 0.001, two-way ANOVA).

The peak of the cross-correlation function was used to quantify the reproducibility of the neuronal response during repetitive stimulation (inset Figures 5A,D; Joris et al., 2006). BCs and SCs displayed a similar variability of neuronal responses which increased with age at *F*_mod_ = 20 Hz and 50 Hz (*F*_mod_ = 20 Hz; effect of cell type *p* = 0.44, effect of age *p* = 0.02, interaction cell type × age *p* = 0.65; *F*_mod_ = 50 Hz; effect of cell type *p* = 0.88, effect of age *p* = 0.01, interaction cell type × age *p* = 0.78; two-way ANOVA). Similar to the modulation depth at *F*_mod_ = 100–500 Hz, reproducibility did not change with maturity (*F*_mod_ = 100 Hz: effect of cell type *p* = 0.93, effect of age *p* = 0.59, interaction cell type × age *p* = 0.58; *F*_mod_ = 200 Hz: effect of cell type *p* = 0.96, effect of age *p* = 0.46, interaction cell type × age *p* = 0.13; *F*_mod_ = 500 Hz: effect of cell type *p* = 0.03, effect of age *p* = 0.83, interaction cell type × age *p* = 0.1; two-way ANOVA). Notably, BCs showed a higher reproducibility at *F*_mod_ = 500 Hz indicating consistent neuronal responses to transient changes in stimulus envelopes. In summary, responses to SAM stimulation revealed that both BCs and SCs can follow amplitude modulations immediately after hearing onset. While at low *F*_mod_ the reproducibility values decreased with maturity, probably due to generally higher firing adding a stochastic component to the stimulus-evoked spike generation, BCs and SCs respond to SAM stimulation at high *F*_mod_ in a similar manner throughout development. In addition, BCs have a higher VS, deeper modulation and higher reproducibility of responses than SCs at 500 Hz *F*_mod_.

## Discussion

The present study investigates the functional maturation of BCs and SCs, the two principal neuron types in the AVCN that encode different sound features in segregated afferent brainstem pathways. To date, the *in vivo* development of signal processing during the early auditory experience in the AVCN of mice remained elusive. We employed single-unit juxtacellular recordings in anesthetized CBA/J mice to investigate whether the maturation time course of BCs and SCs follows a cell-specific pattern, despite both receiving primary excitatory inputs from ANFs. The results demonstrate that the cochlear maturation initially determines the response properties after hearing onset, as seen from threshold- and CF-values, which in both cell types promptly develop by P14. In BCs, acoustic information processing is mature-like by P18 regarding maximal firing rate, dynamic range, PSTH, recovery from post-stimulus depression, FSL, and SAM encoding. In SCs, however, the pattern of spontaneous activity, response properties to acoustic stimulation, and the maximal firing rate show prolonged maturation beyond P18. The effects of acoustically evoked inhibition on frequency response areas were consistently stronger in SCs, probably due to an imbalanced interaction with a still immature excitation. Together, these data suggest that maturation of auditory processing in the two parallel auditory streams engages distinct mechanisms at the first central synapses, differently depending on early auditory experience.

### Peripheral and Central Mechanisms Contribute to Prolonged Development of Auditory Processing in BCs and SCs

AP activity in BCs and SCs depends on the maturation of auditory periphery and the pre- and postsynaptic constituents of AVCN synapses, i.e., auditory nerve synaptic terminals (Fekete et al., 1984; Brown and Ledwith, 1990), postsynaptic receptors (Bellingham et al., 1998; Brenowitz and Trussell, 2001; Lu and Trussell, 2007) and ion channels (Wu and Oertel, 1987; Perney and Kaczmarek, 1997; Bortone et al., 2006). The decrease in response threshold and the extension of hearing range to higher frequencies showed a similar developmental time course for BCs and SCs, reaching mature-like levels already by P14. The increased sensitivity can be attributed to structural development of the middle and inner ear (Kraus and Aulbach-Kraus, 1981; Huangfu and Saunders, 1983), development of the endocochlear potential (Rybak et al., 1992), and the increase in outer hair cell transducer current (Kennedy et al., 2003). The delayed responsiveness to high-frequency stimuli is consistent with the initial maturation of the low-to-middle frequency region in the cochlea, followed by the high-frequency domain (Rübsamen, 1992). The staggered development of other response properties in BCs, and particularly in SCs, suggests that the rapid maturation of the auditory periphery is pace-setting for the development of adult-like threshold and frequency representation by P14.

Between P12 and P30, the FSL shortens by ~34% in BCs (2.1 ms) and by ~30% in SCs (2.5 ms). During this period, the transmission delay, measured as prepotential-AP time in BCs, shortened by ~0.3 ms. Shortening of EPSP latency in AVCN units of mice had also been observed in slice recordings (Wu and Oertel, 1987). Yet, maturation of the endbulb of Held-BC synapses can only partially account for the prominent reduction in FSL. Therefore, faster AP responses are presumably determined by fast synaptic transmission between IHCs and spiral ganglion neurons (Beurg et al., 2010; Grant et al., 2010) and increased conduction velocity of auditory nerve fibers due to the progressive myelination (Ryugo et al., 2006). In addition, the development of intrinsic membrane properties and increasing internodal distances could potentially contribute to faster firing responses.

### Development of Auditory Processing Follows a Cell-Type Specific Time Course

Spontaneous and acoustically evoked firing rates were adult-like by P18 in BCs, whereas SCs showed further maturational changes up to P30. Increased spontaneous firing after hearing onset can be explained by a gradual recruitment of auditory nerve fibers with low threshold and high spontaneous rates (Romand, 1984; Walsh and McGee, 1987; Wu et al., 2016), mediated by accumulation of Ca^2+^ channels at the respective IHC active zone (Walsh and McGee, 1987; Wong et al., 2013; Wu et al., 2016). However, the differences in maturation of firing activity between cell types indicate that synaptic morphology, physiology, and intrinsic properties have different developmental dynamics. Patterned spontaneous activity generated by the immature IHCs (Glowatzki and Fuchs, 2002; Tritsch et al., 2010) was recorded in 46% of P14 SCs, while 50% of BCs showed solely Poisson-like firing already at P12. In line with this, the mature-like chopping response to acoustic stimulation was recorded in only about 40% of ≤P14 SCs. Several factors could account for this: (i) Extended development of the NMDA component up to P17, which is required to endow SCs with slow EPSCs (Cao and Oertel, 2010). (ii) Although quantal size and mEPSC kinetics do not change between P7 and P21 (Lu et al., 2007), it is possible that the readily releasable vesicle pool grows and the release probability decreases, thereby increasing synaptic efficacy at the auditory nerve-SC terminals, similar to the developing calyx of Held (Taschenberger and von Gersdorff, 2000; Iwasaki and Takahashi, 2001). This is corroborated by the data showing smaller synaptic-depression and increased efficacy between P7 and P22 SCs (Wu and Oertel, 1987). (iii) The upregulation of Kv3.1 between P3 and P21 has been demonstrated to allow for fast APs, thus enabling rapid, repetitive firing of adult SCs (Perney and Kaczmarek, 1997; Rothman and Manis, 2003; Bortone et al., 2006; Friedland et al., 2007). The potassium conductance in SCs is dominated by K_v_3.3, over K_v_3.1, while K_v_1 are not expressed, which can also explain consistently faster APs in SCs compared to BCs (Perney and Kaczmarek, 1997; Rothman and Manis, 2003; Caminos et al., 2005; Friedland et al., 2007).

The intrinsic properties of BCs are seemingly mature by P18, which can be concluded from the stable AP kinetics up to P30. The increasing postnatal expression of Kv1.1. and Kv1.2, the two dominant potassium channels in BCs (Bortone et al., 2006), and their redistribution from the cell body to axon (Fitzakerley et al., 2000; Bortone et al., 2006) probably account for early maturation of AP kinetics. However, faster synaptic transmission between P18 and P30 suggests changes in the presynaptic release machinery and/or postsynaptic receptors as well. Recordings from P9–11 mouse endbulbs showed rapid APs that release about 10% of vesicles from a readily releasable pool estimated at about 1,000 vesicles (Lin et al., 2011). Similar to the calyx of Held (Fedchyshyn and Wang, 2005; Nakamura et al., 2015), a tighter coupling of synaptic vesicles to Ca^2+^-channels causing faster release could contribute to the shortening of the transmission delay. Prolonged structural changes at the endbulb, extending to the second postnatal month in mice (Limb and Ryugo, 2000) indicate that functional development may also extend well after hearing onset.

The reciprocal development of postsynaptic AMPA and NMDA receptors renders brief mEPSCs and eEPCSs at calyceal synapses (Futai et al., 2001; Joshi et al., 2004; Lu and Trussell, 2007). The increase in AMPAR conductance after hearing onset (Bellingham et al., 1998; Cao and Oertel, 2010), mediated by GluR3 and GluR4 isoforms expressed as flop splice variants (Wang et al., 1998; Gardner et al., 2001), enables rapid gating and brief mEPSCs with maturity (Lu et al., 2007). Together with putative presynaptic changes, the maturation of postsynaptic receptors probably contributes to speeding of synaptic transmission at the endbulb-BC synapse.

Encoding of stimulus envelope was comparable between BCs and SCs for *F*_mod_ = 20–200 Hz. The presently recorded chopper units were well tuned to lower *F*_mod_, emphasizing the role of SCs in detection of low-frequency envelope modulation (Rhode and Greenberg, 1994a; Joris et al., 2004; Sayles et al., 2013), which is essential for the processing of complex communication signals (Smith et al., 2002), including speech in humans (Shannon et al., 1995; Smith et al., 2002). Still, primary like units, i.e., BCs were better in transmitting envelope information at higher modulation frequency (*F*_mod_ = 500 Hz), consistent with a role in preserving temporal fine structure cues (Frisina et al., 1990; Joris et al., 1994; Keine et al., 2017; Paraouty et al., 2018). The timing information encoded by phasic onset firing of BCs is crucial for extraction of interaural signal differences underlying both, spatial hearing and the perception of tonal pitch (Palmer et al., 1986; Carr, 1993; Smith et al., 1993; Pijl and Schwarz, 1995; Paolini et al., 2001). Although BCs and SCs continue to mature after hearing onset, the processing of rapid amplitude modulations, presently explored by SAM stimulation, seems to be established right after hearing onset. However, an in depth analysis will be required to investigate potential developmental differences between the cell types regarding the processing of complex natural sounds.

### Integration of Excitation and Inhibition During Development

Starting with hearing onset, acoustically evoked inhibition, particularly effective at the high- and low-frequency flanks of the excitatory response areas of chopper units contributed to the sharp tuning (Figure 3). These neurons, presently referred to as SCs are consistent with T-SCs as classified by Smith and Rhode (1989). This indicates that acoustically evoked inhibition presumably matures in parallel to the respective excitation, and initially might (at least in part) even prevail excitation. This can be concluded from the prolonged development up to P30 of the units’ maximal firing rates and rate level functions. Still, this does not exclude the possibility of further maturation of inhibitory signaling after hearing onset. Spectrally broad inhibition is likely mediated by glycinergic D-SCs from within the AVCN (Xie and Manis, 2013; Campagnola and Manis, 2014). In addition, SCs receive narrowband inhibition from glycine-containing tuberculoventral cells in the dorsal cochlear nucleus (DCN; Wickesberg and Oertel, 1990; Zhang and Oertel, 1993; Campagnola and Manis, 2014). Inhibitory inputs to SCs elicit brief IPSCs that can block redundant spikes triggered by slow NMDA currents, thereby improving encoding of envelope cues which facilitates target detection in the presence of modulated maskers (Pressnitzer et al., 2001; Xie and Manis, 2013).

The characteristic high-frequency inhibitory sidebands in the frequency response area of BCs (F_inh_ in Figure 3; Winter and Palmer, 1990; Rhode and Greenberg, 1994b; Paolini et al., 2005; Typlt et al., 2012; Nerlich et al., 2014b; Keine and Rübsamen, 2015) developed gradually after hearing onset (1/7 at P12 and 2/9 at P13 units with inhibitory sideband). The gross inhibitory inputs on BCs and SCs apparently originate from the same sources (Wickesberg and Oertel, 1990; Saint Marie et al., 1991; Campagnola and Manis, 2014), thus providing broadly tuned inhibition also to BCs (Kuenzel et al., 2011; Keine et al., 2016). Synaptogenesis of glycinergic terminals and the development of the release machinery continue after hearing onset, while the number of postsynaptic GABA_A_R seemingly decreases (Luján et al., 2008; Nerlich et al., 2017). Thus, mature IPSCs are predominantly mediated by glycine receptors, having slower kinetics compared to SCs (Xie and Manis, 2013; Nerlich et al., 2014a,b). The activity-dependent build-up of inhibitory conductance provides a slow onset and offset gain control (Nerlich et al., 2014b; Keine and Rübsamen, 2015) that shapes BC responses in terms of tuning (Caspary et al., 1994; Gai and Carney, 2008), monotonicity of the rate level function (Winter and Palmer, 1991; Kopp-Scheinpflug et al., 2002; Kuenzel et al., 2015; Keine et al., 2016), improvement of phase-locking (Joris et al., 1994; Dehmel et al., 2010; Kuenzel et al., 2011), and reproducibility of coding through enhancement of temporal information at the cost of sound level representation (Keine et al., 2016, 2017).

In summary, temporal processing of auditory information, required for the sound source localization in the superior olivary complex, is largely mature by P18 in BCs. Response features of SCs partially mature up to P30, due to staggered development of excitation. This implies that early auditory experience may differently contribute to development/refinement of calyceal synapses on BCs, and conventional bouton synapses on SCs.

## Data Availability

The datasets for this manuscript are not publicly available because the data is stored on institute’s server and will be made available upon request to any interested party. Requests to access the datasets should be directed to ivan.milenkovic@uni-oldenburg.de.

## Author Contributions

IM, SJ and RR conceived the experiments and wrote the manuscript. MM, SJ, CK, and TR acquired and analyzed the data. CK revised the manuscript critically for important intellectual content. IM and RR provided funding. All authors approved the final version of the manuscript, and agree to be accountable for all aspects of the work in ensuring that questions related to the accuracy or integrity of any part of the work are appropriately investigated and resolved. All persons designated as authors qualify for authorship, and all those who qualify for authorship are listed.

## Conflict of Interest Statement

The authors declare that the research was conducted in the absence of any commercial or financial relationships that could be construed as a potential conflict of interest.
